# Simultaneous Hetero- and Isovalent Doping as the Strategy for Improving Transport Properties of Proton Conductors Based on BaLaInO_4_

**DOI:** 10.3390/ma14216240

**Published:** 2021-10-20

**Authors:** Nataliia Tarasova, Anzhelika Galisheva, Irina Animitsa, Ksenia Belova

**Affiliations:** The Institute of High Temperature Electrochemistry of the Ural Branch of the Russian Academy of Sciences, 620066 Ekaterinburg, Russia; a.o.galisheva@urfu.ru (A.G.); irina.animitsa@urfu.ru (I.A.); ksenia.belova@urfu.ru (K.B.)

**Keywords:** BaLaInO_4_, layered perovskite, Ruddlesden-Popper structure, water uptake, oxygen-ion conductivity, protonic conductivity

## Abstract

This work focused on the novel electrochemical energy material with significantly improved electrical properties. The novel complex oxide Ba_1.1_La_0.9_In_0.5_Y_0.5_O_3.95_ with layered perovskite structure was obtained for the first time. It was proven that the simultaneous introduction of barium and yttrium ions in the structure of BaLaInO_4_ leads to the increase in the unit cell volume of up to 4% and water uptake by about three times. The increase in the proton conductivity values was both due to an increase in the proton concentration and their mobility. The sample Ba_1.1_La_0.9_In_0.5_Y_0.5_O_3.95_ was a nearly pure proton conductor below 400 °C. The co-doping strategy allowed us to increase the protonic conductivity values up to two orders of magnitude and it is the successful method for the design of novel protonic conductors based on the layered perovskites.

## 1. Introduction

**Figure 1:**
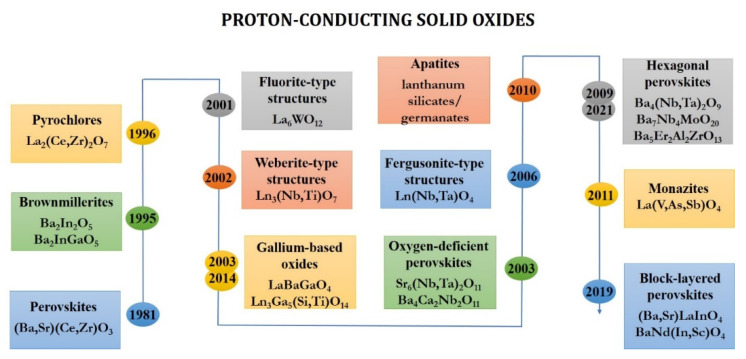
Historical overview on the development of proton-conducting solid oxides.

The proton-conducting solid oxides were revealed for the first time at the early 1980s. The first of these were derivative from SrCeO_3_ and characterized by the perovskite structure [1,2,3]. The progress of materials science research of proton-conducting systems was due to the possibility of using them in the different energy-related applications like solid oxide fuel cell. Today, the problem of creation of high-efficiency, long-term, and low-cost devices is especially relevant due to the need to switchover to clean and renewable energy sources [4,5,6,7]. Over the past forty years, the proton-conducting solid oxides went beyond perovskites family (Figure 1). After variously doped barium cerates and zirconates [8,9,10], classes of proton-conducting materials like hexagonal [11,12,13,14,15,16,17], oxygen-deficient [18,19,20], block-layered [21,22,23,24,25,26,27,28,29,30,31] perovskites, brownmillerites [32,33,34], pyrochlores [35,36,37,38], apatites [39], monazites [40,41,42], gallium-based oxides [43,44,45], materials with fluorite- [46,47,48], fergusonite- [49,50,51], and weberite-types [52,53] of structures were described.

In general, the realization of proton transfer is provided by the dissociative water uptake from the gas phase into the crystal lattice of the solid oxide. However, the crystal structure features determine the sites of protons localization. For oxides like doped barium cerates and zirconates, the proton species localize in the oxygen vacancies that appear through the acceptor doping [54]:

(1) $$V_{o}^{••}+H_{2}O+O_{o}^{\times }↔{2(\mathrm{OH})}_{o}^{•}$$

In such systems, the amount of protons is small and limited by the amount of dopant. The oxygen-deficient perovskites and brownmillerites have oxygen vacancies in the structure without doping up to 0.5 mol per perovskite ABO_3_ formula unit. This leads to a significant increase in the amount of water uptake compared with doped perovskites [54]:

(2) $$V_{o}^{\times }+H_{2}O+{2O}_{o}^{\times }↔{2(\mathrm{OH})}_{o}^{•}+O_{i}^{\prime \prime }$$

In the structure of layered perovskites AA′BO_4_ the alternation of salt layers [AO] and perovskite layers [A′BO_3_] provides the presence of interlayer space in the structure, which is suitable for the insertion of protonic species up to 3 mol theoretically:

(3) $$H_{2}O+O_{o}^{\times }↔{(\mathrm{OH})}_{o}^{•}+{(\mathrm{OH})}_{i}^{\prime }$$

From the point of view of protonic conductivity, the layered perovskites based on BaLaInO_4_, SrLaInO_4_, BaNdInO_4,_ and BaNdScO_4_ were investigated [21,22,23,24,25,26,27,28,29,30,31]. The possibility of acceptor (Ca^2+^/Sr^2+^/Ba^2+^ → La^3+^/Nd^3+^) and donor (Zr^4+^/Ti^4+^/Nb^5+^ → In^3+^) doping was proven. It was shown that a significant amount of protons (up to 1.5 mol) are achieved only for doped compositions, and the increase of dopant concentration leads to the increase in water uptake due to increase in the interlayer space [31]. In the other words, the amount of proton current carriers in the structure increases with an increasing acceptor or dopant content. However, it is well known that the value of protonic conductivity is determined not only by the amount of protonic species but by their mobility as well. The mobility of protons depends on the many factors, including the bond energy with nearest atoms, the possibility to use the migration pathways and the presence of other structural defects. The last factor plays a significant role not only for the classic doped perovskites, but also for the novel block-layer proton conductors. It was shown that the formation of proton-aggregating clusters can be obtained at a small enough amount of acceptor or donor dopant [26,27]:

(4) $$M_{A}^{\prime }+{(\mathrm{OH})}_{o}^{•}\to {\left(M_{A}^{\prime }\cdot {(\mathrm{OH})}_{o}^{•}\right)}^{\times }$$

(5) $$M_{B}^{•}+{(\mathrm{OH})}_{i}^{\prime }\to {\left(M_{B}^{•}\cdot {(\mathrm{OH})}_{i}^{\prime }\right)}^{\times }$$

This means that increase in the acceptor/donor dopant concentration leads to the trapping of protons. Therefore, this way (increase in dopant concentration of M′_A_ or M**˙**_B_) is not prospective for obtaining of block-layered materials with high values of proton conductivity. On the other hand, for layered perovskites, it is known that an increase in the water uptake occurs with an increase in the size of the salt block, and this can be achieved by isovalet doping with a larger ion. Therefore, the strategy of introducing two types of dopants can lead to a more significant increase in proton conductivity.

In this paper, a novel method of improving of proton conductors based on block-layered perovskite BaLaInO_4_ is proposed. The idea of creation of an “optimal” concentration of oxygen vacancies by introducing the acceptor dopant Ba^2+^ → La^3+^ while simultaneously increasing the interlayer space by the isovalent doping Y^3+^ → In^3+^ by ions with bigger ionic radii is realized. The composition of Ba_1.1_La_0.9_In_0.5_Y_0.5_O_3.95_ was obtained for the first time. The possibility for water uptake and realization of fast protonic transport was revealed.

It can be said that the block-layered perovskites are one of the newest classes among proton-conducting solid oxides and require further investigation.

## 2. Materials and Methods

**Figure 2:**
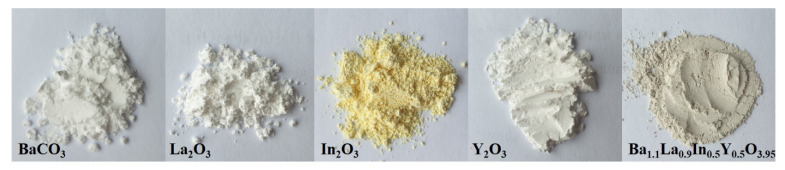
The pictures of powders of initial materials and obtained composition.

**Figure 3:**
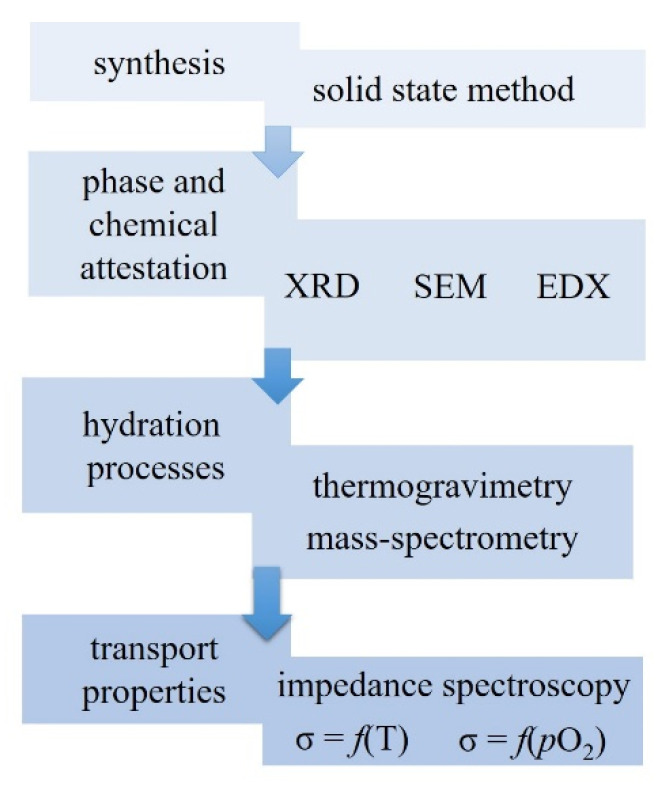
The scheme indicating the workflow and analysis.

The composition of Ba_1.1_La_0.9_In_0.5_Y_0.5_O_3.95_ was prepared by a solid-state method. The initial powders of BaCO_3_, La_2_O_3_, In_2_O_3_, Y_2_O_3_ were dried and weighed using Sartorius balances. The reagents were milling in an agate mortar and then calcined at 800–1300 °C (step of 100 °C, time of calcined 24 h). Figure 2 provides the pictures of the powders of the initial materials and the obtained composition. Figure 3 represents the scheme indicating the workflow and analysis. 

The monitoring of the phase purity of the samples was performed using a Bruker Advance D8 Cu K*_α_* diffractometer with Cu Kα radiation in the range of 2θ = 10 − 80° with a step of 0.01° and at a scanning rate of 0.5°/min. The scanning electron microscope JEOL JSM–6390 LV (WD 8.1 mm, aperture size 30 μm, EHT 20 kV, system vacuum 1.68×10^−6^ mbar) was used to determine the morphology and chemical composition of the samples.

Thermogravimetry (TG) and mass-spectrometry (MS) analysis were performed using the STA 409 PC Netzsch Analyser coupled with a QMS 403 C Aëolos mass spectrometer. The preliminarily hydrated samples were heated at the rate of 10 °C/min in a corundum crucible under a flow of dry Ar at the temperature range of 40–1100 °C. The hydrated forms of the samples were prepared by slow cooling (1 °C/min, 1100–150 °C) in wet Ar.

The impedance spectroscopy measurements were performed on the pressed cylindrical pellets (1300 °C, 24 h) using a Z-1000P (Elins, RF) impedance spectrometer. The measurements were carried out in the temperature range 200–1000 °C every 10–20 °C with a cooling rate of 1°/min under dry and wet air or Ar. Dry gas was obtained by the circulation through P_2_O_5_ (*p*H_2_O = 3.5 × 10^−5^ atm). Wet gas was produced by bubbling first through distilled water and then through saturated solution of KBr (*p*H_2_O = 2×10^−2^ atm). The conductivity was also measured at different partial oxygen pressures *p*O_2_ for some temperatures. The *p*O_2_ was controlled by electrochemical method. The oxygen pump (and sensor) from Y-stabilized ZrO_2_ ceramic was used to control (and measure) *p*O_2_. Before the measurements, the samples were equilibrated up to constant values of resistance for 3–5 h.

## 3. Results and Discussion

### 3.1. X-ray, SEM and TG Characterization

**Figure 4:**
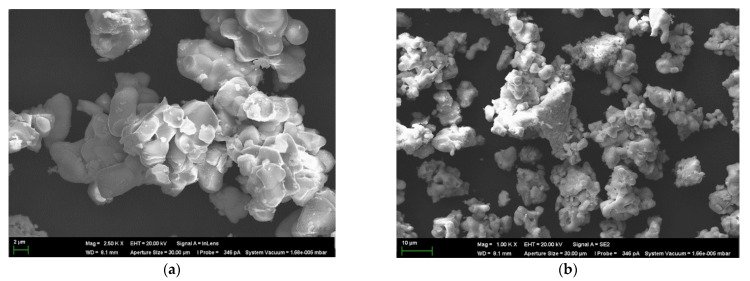
The SEM-images of the composition Ba_1.1_La_0.9_Y_0.5_In_0.5_O_3.95_ (**a**,**b**)

The phase composition of the obtained sample Ba_1.1_La_0.9_In_0.5_Y_0.5_O_3.95_ was checked via scanning electron microscopy (SEM) coupled with energy dispersive analysis (EDS). Figure 4 shows the morphology of the powder sample. The grains of co-doped sample are round-shaped (~3–5 μm) and form the agglomerates of 10–20 μm with an irregular shape. An element analysis was performed at the polished cleavages of the ceramic samples via the EDS method. The experimental content of the elements was in good agreement with the theoretical values (Table 1). 

**Figure 5:**
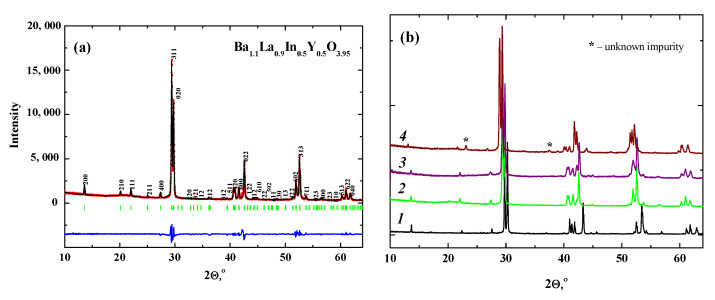
The refinement of XRD-data for the composition Ba_1.1_La_0.9_Y_0.5_In_0.5_O_3.95_ (**a**) and the XRD-data for BaLaInO_4_ (1), Ba_1.1_La_0.9_Y_0.5_In_0.5_O_3.95_ before (2) and after (3) electrochemical measurements and for Ba_1.1_La_0.9_Y_0.6_In_0.4_O_3.95_ (4) (**b**).

The XRD pattern of the co-doped sample Ba_1.1_La_0.9_In_0.5_Y_0.5_O_3.95_ is shown in Figure 5. (Figure 5a). It is observed that all reflexes belong to the phase with orthorhombic symmetry, so the sample exhibits an RP-structure with the *Pbca* space group in agreement with the XRD data for the parent phase BaLaInO_4_ [24]. The cell parameters for undoped BaLaInO_4_ [24] and doped Ba_1.1_La_0.9_InO_3.95_ [24], Ba_1.1_La_0.9_In_0.5_Y_0.5_O_3.95_ samples are shown in Table 2. As it was shown earlier, the acceptor doping of lanthanum sublattice Ba^2+^ → La^3+^ led to an increase in all unit cell parameters and the unit cell volume. The simultaneous doping Ba^2+^ → La^3+^ and Y^3+^ → In^3+^ leads to the same effect, but the increase in the unit cell volume during co-doping is bigger (~4%) compared with increase in the case of only acceptor doping (~1%). It should be noted that the compositions with a yttrium content over 50% were not singe phases (Figure 5b, line 4). After measuring the conductivity of the synthesized phase Ba_1.1_La_0.9_In_0.5_Y_0.5_O_3.95_ as a function of pO_2_ at different temperatures and high humidity, X-ray powder diffraction patterns of the pellets were obtained (Figure 5b. line 3). Decomposition, due to the highly reduced atmosphere or to the high humidity to which the materials were exposed, did not take place.

**Figure 6:**
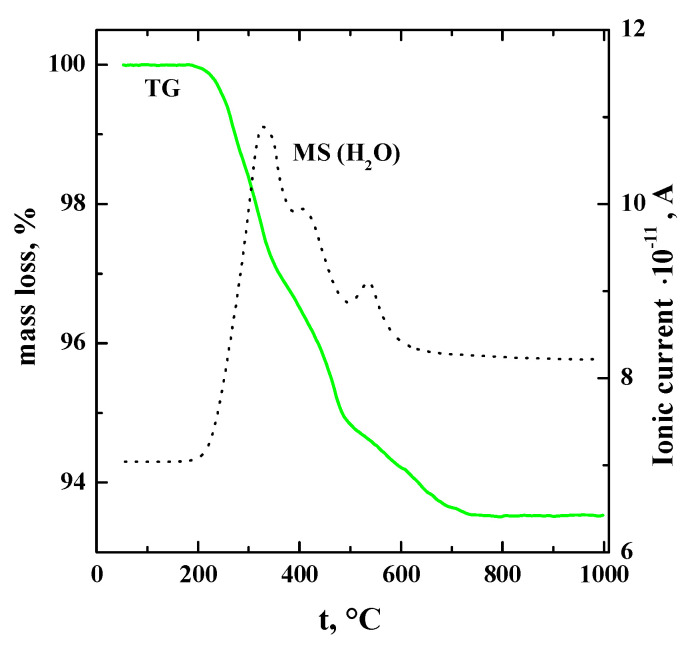
The TG− and MS−data for the composition Ba_1.1_La_0.9_Y_0.5_In_0.5_O_3.95._

The possibility for the dissociative water incorporation from the gas phase was checked using the thermogravimetry (TG) method. The mass loss of preliminary hydrated sample Ba_1.1_La_0.9_In_0.5_Y_0.5_O_4.95_∙*n*H_2_O was 93.5% (Figure 6). The mass spectrometry (MS) analysis showed that all mass loss was a result of only water removal. The calculated amount of mole of water per the formula unit is presented in the Table 2. As we can see, the water uptake increases with the increasing unit cell volume. As shown earlier [31], the amount of water uptake for layered perovskites based on BaLaInO_4_ is determined not by the amount of oxygen vacancies but by the size of unit cell volume. The result obtained for the co-doped sample Ba_1.1_La_0.9_In_0.5_Y_0.5_O_3.95_ is in agreement with this statement.

### 3.2. Electrical Properties

**Figure 7:**
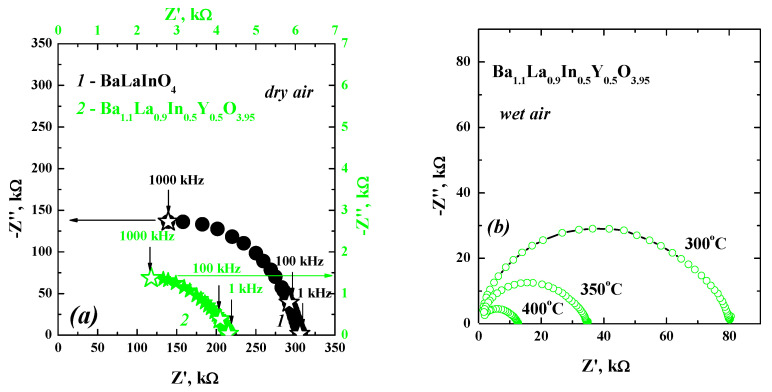
The Nyquist plots for the compositions BaLaInO_4_ [24] (1) and Ba_1.1_La_0.9_Y_0.5_In_0.5_O_3.95_ (2) obtained at 500 °C under dry air (**a**) and for the composition Ba_1.1_La_0.9_Y_0.5_In_0.5_O_3.95_ obtained at different temperatures under wet air (**b**).

The electrical properties of the obtained sample were investigated via the impedance spectroscopy method. The comparison of Nyquist plots for undoped BaLaInO_4_ and doped Ba_1.1_La_0.9_In_0.5_Y_0.5_O_3.95_ samples is presented in Figure 7a (500 °C, dry air). As can be seen, the doping does not affect to the general view of the plots. The one semicircle starting from zero coordinates and corresponding to the bulk component (C_bulk_~10^–11^ F∙cm^–1^) of conductivity is observed for both samples. The bulk conductivity value for the doped sample Ba_1.1_La_0.9_In_0.5_Y_0.5_O_3.95_ is about 4 kΩ at 500 °C in the dry air (green symbols in Figure 7a) which is higher by almost two orders of magnitude than for the undoped sample (300 kΩ, black symbols in Figure 7a). As an example of the evolution of spectra, the Nyquist plots obtained in the wet air for the composition Ba_1.1_La_0.9_In_0.5_Y_0.5_O_3.95_ are presented in Figure 7b. 

**Figure 8:**
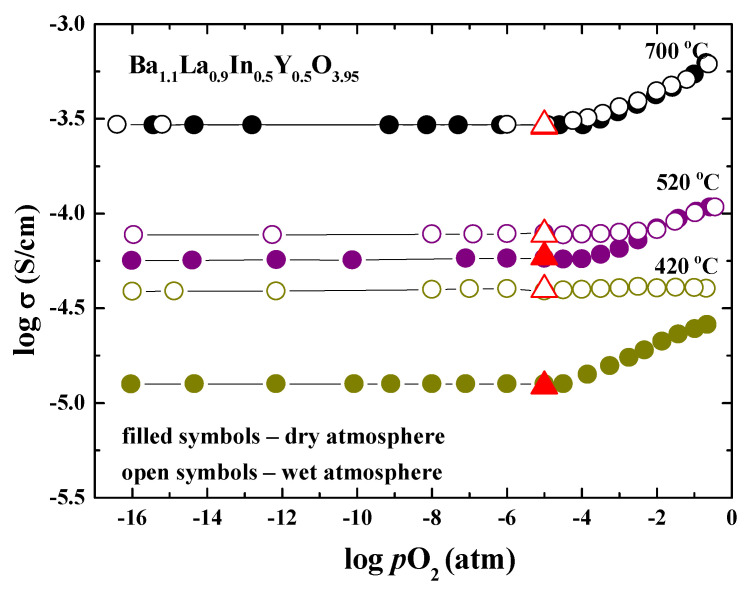
The total conductivity vs. *p*O_2_ for the composition Ba_1.1_La_0.9_Y_0.5_In_0.5_O_3.95_ at dry (filled symbols) and wet (open symbols) conditions; and conductivity values from *σ*–10^3^/*T* dependencies at dry Ar (red symbols).

The nature of charge carriers was discovered by investigating conductivity through the variation of oxygen partial pressure. The dependencies of conductivity vs. *p*O_2_ at different temperatures for the doped sample Ba_1.1_La_0.9_In_0.5_Y_0.5_O_3.95_ are presented in Figure 8. The positive slope of the conductivity curves obtained in the dry oxidizing conditions (*p*O_2_ = 10^−5^ − 0.21 atm) indicates the mixed ionic-electronic (hole) nature of conductivity:

(6) 12O2↔Oi″+2h•

The electrolytic area (*p*O_2_ = 10^−16^ − 10^−5^ atm) is characterized by an independence of conductivity values from oxygen partial pressure which indicates the domination of oxygen-ionic conductivity in this *p*O_2_ region. The effect of humidity in the atmosphere is shown to start below 700 °C. The conductivity values increase in the electrolytic area up to half an order of magnitude at 420 °C, which indicates the appearance of proton current carriers (Equation (3)). In the area of wet oxidizing conditions, the interaction of holes with water leads to the formation of protons:

(7) h•+12H2O+Oi″↔14O2+(OH)i′

**Figure 9:**
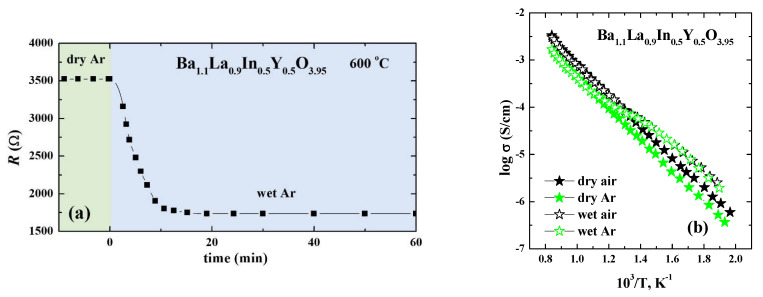
The resistance as a function of exposure time for the sample Ba_1.1_La_0.9_Y_0.5_In_0.5_O_3.95_ obtained during switched from dry Ar to wet Ar atmosphere (**a**), and the temperature dependencies of conductivities for the composition Ba_1.1_La_0.9_Y_0.5_In_0.5_O_3.95_ obtained under dry (open symbols) and wet (filled symbols) air (black symbols) and Ar (green symbols) (b).

The effect of humidity of atmosphere on the transport properties was investigated at the different temperatures. The example of initial data is presented in the Figure 9a. During the measurement, the sample was held at each temperature until the resistances became constant. It can be seen that the switching from dry to wet atmosphere leads to a decrease in the resistance values, i.e., to an increase in the values of electrical conductivity. The temperature dependencies of conductivity for the doped sample Ba_1.1_La_0.9_In_0.5_Y_0.5_O_3.95_ obtained for the different conditions are presented in Figure 9b. The conductivity values in the dry air (filled black symbol) were higher than that in the dry Ar (filled green symbol) by about 0.3 order of magnitude. This indicates the mixed ionic-electronic nature of conductivity in the whole temperature range. An increase in the humidity of the atmosphere led to an increase in the conductivity values compared with dry conditions. The conductivity values in the wet air (open black symbol) and wet Ar (open green symbol) were the same at temperatures lower than 400 °C, indicating the domination of proton transport in this area. It should be noted that the conductivity values obtained in the Ar (*p*O_2_~10^−5^ atm) were matched with the values from the electrolytic area (red symbols in Figure 8). Thus, the values obtained in the dry Ar can be considered as oxygen-ionic conductivity values. 

**Figure 10:**
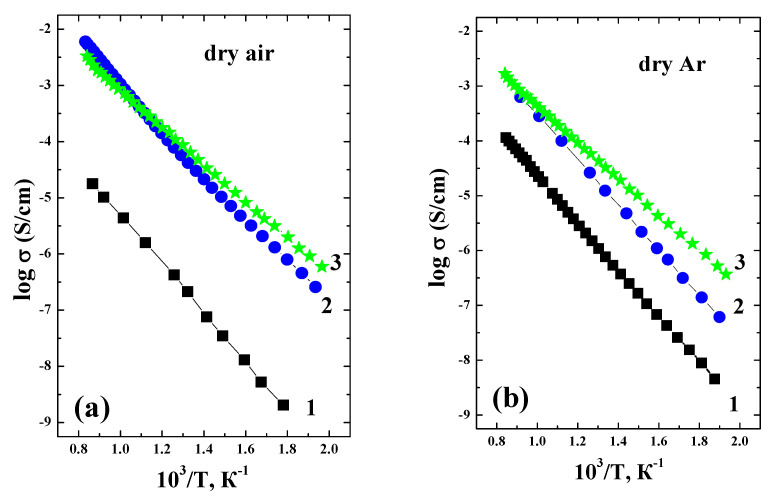

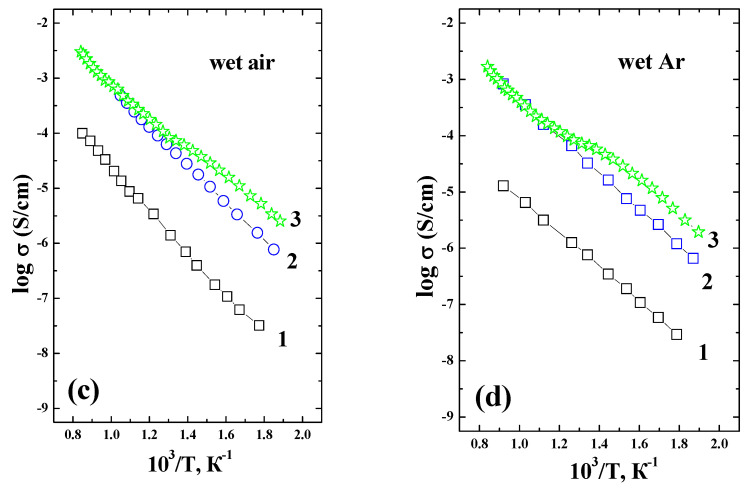
The temperature dependencies of conductivities for the BaLaInO_4_ [24] (1), Ba_1.1_La_0.9_InO_3.95_ [24] (2), and Ba_1.1_La_0.9_In_0.5_Y_0.5_O_3.95_ (3) obtained under dry air (**a**), dry Ar (**b**), wet air (**c**), wet Ar (**d**).

Figure 10 represents the comparison of temperature dependencies of conductivity obtained for the different conditions (dry/wet air and Ar). As can be seen, the conductivity increases in the row BaLaInO_4_–Ba_1.1_La_0.9_InO_3.95_–Ba_1.1_La_0.9_In_0.5_Y_0.5_O_3.95_ for all conditions, i.e., in the row of an increasing unit cell volume and interlayer space (space between perovskite octahedra layers). It was shown that ionic transport for layered perovskites like BaNdInO_4_ [22] and BaLaScO_4_ [23] occurs via oxygen jumps between apical oxygens in the perovskite octahedra and oxygens in the salt layers, i.e., in the interlayer space of the layered structure. We can suggest that the same mechanism of oxygen transport is realized for the layered perovskites based on BaLaInO_4_. 

**Figure 11:**
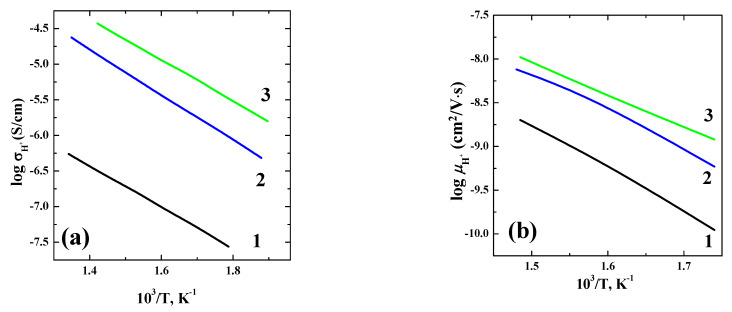
The concentration dependencies of protonic conductivity (**a**) and protonic mobility (**b**) for the composition Ba_1.1_La_0.9_Y_0.5_In_0.5_O_3.95._

The protonic conductivity values were calculated as the difference between values obtained in the wet and dry Ar at the same temperature (Figure 11a). The tendency for conductivity to increase with the increasing unit cell volume is reproduced. However, in the row BaLaInO_4_–Ba_1.1_La_0.9_InO_3.95_–Ba_1.1_La_0.9_In_0.5_Y_0.5_O_3.95_ both the size of the interlayer space and the proton concentration (water uptake) increase. Accordingly, calculating the proton mobility for a correct comparison is required:

*μ*_H_ = *σ*_H_/*Zec*_H_, (8)

where *σ*_H_–proton conductivity, *Ze*–charge (*Z* = 1), *c*_H_–volume concentration of protons. As can be seen in Figure 11b, the protonic mobility increases with increasing the size of interlayer space, i.e., with increasing the space for ionic transport. 

Therefore, the simultaneous doping of barium and indium sublattice by cations with bigger ionic radii leads to the increase of both unit cell volume and water uptake. However, the increasing the proton conductivity values occurs not only by an increase in the proton concentration but by an increase in their mobility as well. This allows us to conclude that the method of simultaneous hetero- and isovalent doping is a very prospective strategy for improving the transport properties of proton conductors with layered perovskite structures.

## 4. Conclusions

In the present work, the method of simultaneous hetero- and isovalent doping for the purpose of improving the transport properties of layered perovskites was investigated. The co-doped complex oxide Ba_1.1_La_0.9_In_0.5_Y_0.5_O_3.95_ was prepared for the first time. It was shown that the co-doping by ions with bigger ionic radii allows for increasing the unit cell volume by up to 4% and water uptake by three times compared with an undoped BaLaInO_4_ composition. The correlation of the increase in ionic conductivity with an increase in the unit cell volume and the interlayer space for layered perovskites was confirmed. The increase in the proton conductivity values obtained was both due to the increase in the proton concentration and their mobility. The sample Ba_1.1_La_0.9_In_0.5_Y_0.5_O_3.95_ was nearly a pure proton conductor below 400 °C. The co-doping strategy allows for increasing the protonic conductivity values by up to two orders of magnitude and it is a successful method for the design of novel protonic conductors based on layered perovskites.

## Figures and Tables

**Table 1:** The average element ratios determined by EDS analysis for the sample Ba_1.1_La_0.9_In_0.5_Y_0.5_O_3.95._

Element	Content of the Elements, Atomic %	
	Theoretical	Experimental
Ba	15.8	15.1
La	12.9	11.4
In	7.2	6.9
Y	7.2	6.8
O	56.9	59.8

**Table 2:** The lattice parameters, unit cell volume and amount of water uptake for the samples BaLaInO_4_ [24], Ba_1.1_La_0.9_InO_3.95_ [24], Ba_1.1_La_0.9_Y_0.5_In_0.5_O_3.95._

Sample	a, Å	b, Å	c, Å	Cell Volume, (Å^3^)	Increase of Cell Volume, %	Water Uptake
BaLaInO_4_	12.932(3)	5.906(0)	5.894(2)	450.18(8)	−	0.62
Ba_1.1_La_0.9_InO_3.95_	13.002(1)	5.919(3)	5.901(3)	454.18(8)	0.89	1.05
Ba_1.1_La_0.9_Y_0.5_In_0.5_O_3.95_	13.059(6)	5.997(5)	5.993(5)	469.44(0)	4.28	1.90

## Data Availability

Not applicable.

## References

[1] Iwahara H., Esaka T. (1981). Proton conduction in sintered oxides and its application to steam electrolysis for hydrogen production. Solid State Ion..

[2] Iwahara H., Uchida H., Maeda N. (1982). High temperature fuel and steam electrolysis cells using proton conductive solid electrolytes. J. Power Sources.

[3] Iwahara H., Uchida H., Tanaka S. (1983). High temperature type proton conductors based on SrCeO3 and its application to solid electrolyte fuel cells. Solid State Ion..

[4] Medvedev D.A. (2021). Current drawbacks of proton-conducting ceramic materials: How to overcome them for real electrochemical purposes. Curr. Opin. Green Sustain. Chem..

[5] Kim J., Sengodan S., Kim S., Kwon O., Bu Y., Kim G. (2019). Proton conducting oxides: A review of materials and applic.ations for renewable energy conversion and storage. Renew. Sustain. Energy Rev..

[6] Abdalla A.M., Hossain S., Nisfindy O.B., Azad A.T., Dawood M., Azad A.K. (2018). Hydrogen production, storage, transportation and key challenges with applications: A review. Energy Convers. Manag..

[7] Hossain S., Abdalla A.M., Jamain S.N.B., Zaini J.H., Azad A.K. (2017). A review on proton conducting electrolytes for clean energy and intermediate temperature-solid oxide fuel cells. Renew. Sustain. Energy Rev..

[8] Iwahara H., Yajima T., Hibino T., Ozaki K., Suzuki H. (1993). Protonic conduction in calcium, strontium and barium zirconates. Solid State Ion..

[9] Iwahara H., Yajima T., Ushida H. (1994). Effect of ionic radii of dopants on mixed ionic conduction (H^+^+O^2-^) in BaCeO_3_-based electrolytes. Solid State Ion..

[10] Yamazaki Y., Hernandez-Sanchez R., Haile S.M. (2010). Cation non-stoichiometry in yttrium-doped barium zirconate: Phase behavior, microstructure, and proton conductivity. J. Mater. Chem..

[11] Ling C.D., Avdeev M., Kutteh R., Kharton V.V., Yaremchenko A.A., Fialkova S., Sharma N., Macquart R.B., Hoelzel M., Gutmann M. (2009). Phase Transitions, Hydration, and Ionic Conductivity of Ba_4_Nb_2_O_9_. Chem. Mater..

[12] Ling C.D., Avdeev M., Kharton V.V., Yaremchenko A.A., Macquart R.B., Hoelzel M. (2010). Phase Transitions, Hydration, and Ionic Conductivity of Ba_4_Ta_2_O_9_. Chem. Mater..

[13] Dunstan M.T., Pavan A.F., Kharton V.V., Avdeev M., Kimpton J.A., Kolotygin V.A., Tsipis E.V., Ling C.D. (2013). Phase behavior and mixed ionic–electronic conductivity of Ba_4_Sb_2_O_9_. Solid State Ion..

[14] Fop S., Skakle J.M.S., McLaughlin A.C., Connor P.A., Irvine J.T.S., Smith R.I., Wildman E.J. (2016). Oxide Ion Conductivity in the Hexagonal Perovskite Derivative Ba_3_MoNbO_8.5_. J. Am. Chem. Soc..

[15] Fop S., McCombie K.S., Wildman E.J., Skakle J.M.S., Irvine J.T.S., Connor P.A., Savaniu C., Ritter C., McLaughlin A.C. (2020). High oxide ion and proton conductivity in a disordered hexagonal perovskite. Nat. Mater..

[16] Yashima M., Tsujiguchi T., Sakuda Y., Yasui Y., Zhou Y., Fujii K., Torii S., Kamiyama T., Skinner S.J. (2021). High oxide-ion conductivity through the interstitial oxygen site in Ba_7_Nb_4_MoO_20_-based hexagonal perovskite related oxides. Nat. Comm..

[17] Murakami T., Hester J.R., Yashima M. (2020). High Proton Conductivity in Ba_5_Er_2_Al_2_ZrO_13_, a Hexagonal Perovskite-Related Oxide with Intrinsically Oxygen-Deficient Layers. J. Am. Chem. Soc..

[18] Animitsa I., Denisova T., Neiman A., Nepryahin A., Kochetova N., Zhuravlev N., Colomban P. (2003). States of H^+^-containing species and proton migration forms in hydrated niobates and tantalates of alkaline-earth metals with a perovskite-related structure. Solid State Ion..

[19] Animitsa I., Neiman A., Kochetova N., Korona D., Sharafutdinov A. (2006). Chemical diffusion of water in the double perovskites Ba_4_Ca_2_Nb_2_O_11_ and Sr_6_Ta_2_O_11_. Solid State Ion..

[20] Tarasova N., Colomban P., Animitsa I. (2018). The short-range structure and hydration process of fluorine-substituted double perovskites based on barium-calcium niobate Ba_2_CaNbO_5.5_. J. Phys. Chem. Solids.

[21] Troncoso L., Arce M.D., Fernández-Díaz M.T., Mogni L.V., Alonso J.A. (2019). Water insertion and combined interstitial-vacancy oxygen conduction in the layered perovskites La_1.2_Sr_0.8-x_Ba_x_InO_4+δ_. New J. Chem..

[22] Zhou Y., Shiraiwa M., Nagao M., Fujii K., Tanaka I., Yashima M., Baque L., Basbus J.F., Mogni L.V., Skinner S.J. (2021). Protonic Conduction in the BaNdInO_4_ Structure Achieved by Acceptor Doping. Chem. Mater..

[23] Shiraiwa M., Kido T., Fujii K., Yashima M. (2021). High-temperature proton conductors based on the (110) layered perovskite BaNdScO_4_. J. Mater. Chem. A.

[24] Tarasova N., Animitsa I., Galisheva A., Korona D. (2019). Incorporation and Conduction of Protons in Ca, Sr, Ba-Doped BaLaInO_4_ with Ruddlesden-Popper Structure. Materials.

[25] Tarasova N., Animitsa I., Galisheva A., Pryakhina V. (2020). Protonic transport in the new phases BaLaIn_0.9_M_0.1_O_4.05_ (M=Ti, Zr) with Ruddlesden-Popper structure. Solid State Sci..

[26] Tarasova N., Animitsa I., Galisheva A. (2020). Electrical properties of new protonic conductors Ba_1+x_La_1–x_InO_4–0.5x_ with Ruddlesden-Popper structure. J. Solid State Electrochem..

[27] Tarasova N., Galisheva A., Animitsa I. (2020). Improvement of oxygen-ionic and protonic conductivity of BaLaInO_4_ through Ti doping. Ionics.

[28] Tarasova N., Animitsa I., Galisheva A. (2020). Effect of doping on the local structure of new block-layered proton conductors based on BaLaInO_4_. J. Raman Spec..

[29] Tarasova N., Galisheva A., Animitsa I. (2021). Ba^2+^/Ti^4+^- co-doped layered perovskite BaLaInO_4_: The structure and ionic (O^2−^, H^+^) conductivity. Int. J. Hydrog. Energy.

[30] Tarasova N., Animitsa I., Galisheva A. (2021). Spectroscopic and transport properties of Ba- and Ti-doped BaLaInO_4_. J. Raman Spec..

[31] Tarasova N., Animitsa I., Galisheva A. (2021). Effect of acceptor and donor doping on the state of protons in block-layered structures based on BaLaInO_4_. Solid State Comm..

[32] Zhang G.B., Smyth D.M. (1995). Protonic conduction in Ba_2_In_2_O_5_. Solid State Ion..

[33] Schober T., Friedrich J., Krug F. (1997). Phase transition in the oxygen and proton conductor Ba_2_In_2_O_5_ in humid atmospheres below 300 °C. Solid State Ion..

[34] Schober T., Friedrich J. (1998). The oxygen and proton conductor Ba2In2O5: Thermogravimetry of proton uptake. Solid State Ion..

[35] Shimura T., Komori M., Iwahara H. (1996). Ionic conduction in pyrochlore-type oxides containing rare earth elements at high temperature. Solid State Ion..

[36] Omata T., Okuda K., Tsugimoto S., Otsuka-Matsuo-Yao S. (1997). Water and hydrogen evolution properties and protonic conducting behaviors of Ca-doped La2Zr2O7 with a pyrochlore structure, Solid State Ion. Solid State Ion..

[37] Sun W., Fang S., Yan L., Liu W. (2012). Investigation on Proton Conductivity of La_2_Ce_2_O_7_ in Wet Atmosphere: Dependence on Water Vapor Partial Pressure. Fuel Cells.

[38] Besikiotis V., Knee C.S., Ahmed I., Haugsrud R., Norby T. (2012). Crystal structure, hydration and ionic conductivity of the inherently oxygen-deficient La_2_Ce_2_O_7_. Solid State Ion..

[39] Orera A., Slater P.R. (2010). Water incorporation studies in apatite-type rare earth silicates/germinates. Solid State Ion..

[40] Huse M., Norby T., Haugsrud R. (2011). Proton Conductivity in Acceptor-Doped LaVO_4_. J. Electrochem. Soc..

[41] Bjørheim T.S., Norby T., Haugsrud R. (2012). Hydration and proton conductivity in LaAsO_4_. J. Mater. Chem..

[42] Winiarz P., Dzierzgowski K., Mielewczyk-Gryn A., Gazda M., Wachowski S. (2021). High-Temperature Proton Conduction in LaSbO_4_. Chem. Eur. J..

[43] Li S., Schönberger F., Slater P. (2003). La_1-x_Ba_1+x_GaO_4-x/2_: A novel high temperature proton conductor. Chem. Commun..

[44] Schönberger F., Kendrick E., Islam M.S., Slater P.R. (2005). Investigation of proton conduction in La_1-x_Ba_1+x_GaO_4-x/2_and La_1-x_Sr_2+x_GaO_5-x/2_. Solid State Ion..

[45] Bjørheim T.S., Haugsrud R., Norby T. (2014). Protons in acceptor doped langasite, La_3_Ga_5_SiO_14_. Solid State Ion..

[46] Shimura T., Fujimoto S., Iwahara H. (2001). Proton conduction in non-perovskite-type oxides at elevated temperatures. Solid State Ion..

[47] Partin G.S., Korona D.V., Neiman A.Y., Belova K.G. (2015). Conductivity and Hydration of Fluorite-Type La_6-x_WO_12-1.5x_ Phases (x = 0.4; 0.6; 0.8; 1). Russ. J. Electrochem..

[48] Haugsrud R. (2007). Defects and transport properties in Ln_6_WO_12_ (Ln=La, Nd, Gd, Er). Solid State Ion..

[49] Haugsrud R., Norby T. (2006). Proton conduction in rare-earth ortho-niobates and ortho-tantalates. Nat. Mater..

[50] Haugsrud R., Norby T. (2006). High-temperature proton conductivity in acceptor-doped LaNbO_4_. Solid State Ion..

[51] Haugsrud R., Norby T. (2007). High-Temperature Proton Conductivity in Acceptor-Substituted Rare-Earth Ortho-Tantalates LnTaO_4_. J. Am. Ceram. Soc..

[52] Shimura T., Tokiwa Y., Iwahara H. (2002). Protonic conduction in lanthanum strontium aluminate and lanthanum niobate-based oxides at elevated temperatures. Solid State Ion..

[53] Haugsrud R., Risberg T. (2009). Protons in Acceptor-Doped La_3_NbO_7_ and La_3_TaO_7_. J. Electrochem. Soc..

[54] Kochetova N., Animitsa I., Medvedev D., Demin A., Tsiakaras P. (2016). Recent activity in the development of proton conducting oxides for high-temperature applications. RSC Adv..

